# Methylglyoxal and Its Adducts: Induction, Repair, and Association with Disease

**DOI:** 10.1021/acs.chemrestox.2c00160

**Published:** 2022-10-05

**Authors:** Seigmund Wai Tsuen Lai, Edwin De Jesus Lopez Gonzalez, Tala Zoukari, Priscilla Ki, Sarah C. Shuck

**Affiliations:** Department of Diabetes and Cancer Metabolism, Arthur Riggs Diabetes and Metabolism Research Institute, City of Hope Comprehensive Cancer Center, Duarte, California 91010, United States

## Abstract

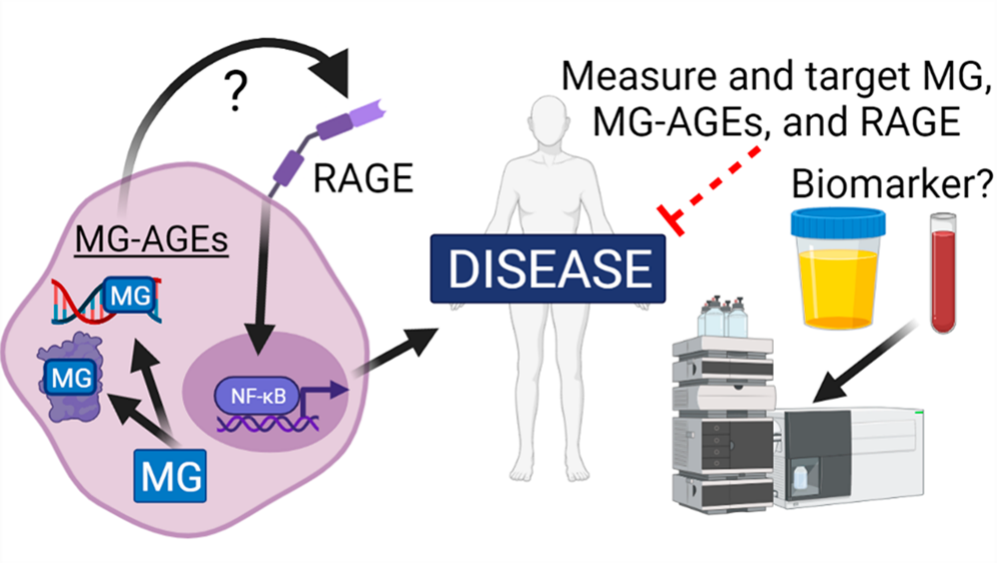

Metabolism is an essential part of life that provides energy for cell growth. During metabolic flux, reactive electrophiles are produced that covalently modify macromolecules, leading to detrimental cellular effects. Methylglyoxal (MG) is an abundant electrophile formed from lipid, protein, and glucose metabolism at intracellular levels of 1–4 μM. MG covalently modifies DNA, RNA, and protein, forming advanced glycation end products (MG-AGEs). MG and MG-AGEs are associated with the onset and progression of many pathologies including diabetes, cancer, and liver and kidney disease. Regulating MG and MG-AGEs is a potential strategy to prevent disease, and they may also have utility as biomarkers to predict disease risk, onset, and progression. Here, we review recent advances and knowledge surrounding MG, including its production and elimination, mechanisms of MG-AGEs formation, the physiological impact of MG and MG-AGEs in disease onset and progression, and the latter in the context of its receptor RAGE. We also discuss methods for measuring MG and MG-AGEs and their clinical application as prognostic biomarkers to allow for early detection and intervention prior to disease onset. Finally, we consider relevant clinical applications and current therapeutic strategies aimed at targeting MG, MG-AGEs, and RAGE to ultimately improve patient outcomes.

## Introduction

Metabolism encompasses all of the reactions cells use to convert food into energy and is essential to sustain life. Changes in metabolic flux result from increased food intake, dysregulation of metabolite uptake, and up- and downregulation of proteins involved in metabolic pathways. Altered metabolism is associated with diabetes, cancer, liver, and kidney disease; however, how this drives disease onset and progression is not clear. A proposed mechanism is through changes in cellular physiology caused by reactive electrophiles produced during metabolic flux. Electrophiles are electron-pair-deficient molecules that react with nucleophilic sites within macromolecules to alter the structure and function. An abundant electrophile produced from metabolic flux is methylglyoxal (MG), which is formed intracellularly at levels of 1–4 μM.^1^ MG covalently modifies nucleophilic sites within nucleic and amino acids, forming advanced glycation end products (MG-AGEs).

Our understanding of the role of MG and MG-AGEs as potential drivers of disease has advanced because of the foundational work by Larry Marnett and other pioneers in the field of chemical toxicology. Marnett described the impact of reactive electrophiles, including malondialdehyde, base propenal, and hydroxynonenal on protein and DNA structure and function.^2−8^ This work provided the framework to investigate the impact of electrophile stress using chemical tools, analytical methods, biochemistry assays, and models for animal studies. Measuring and targeting electrophiles, their associated byproducts, and receptors has important implications as biomarkers and etiological agents of disease. In this review, we discuss the formation of MG, MG-AGEs, the physiological impact of these molecules on cellular function, and their association with disease.

## MG Production

**Figure 1:**
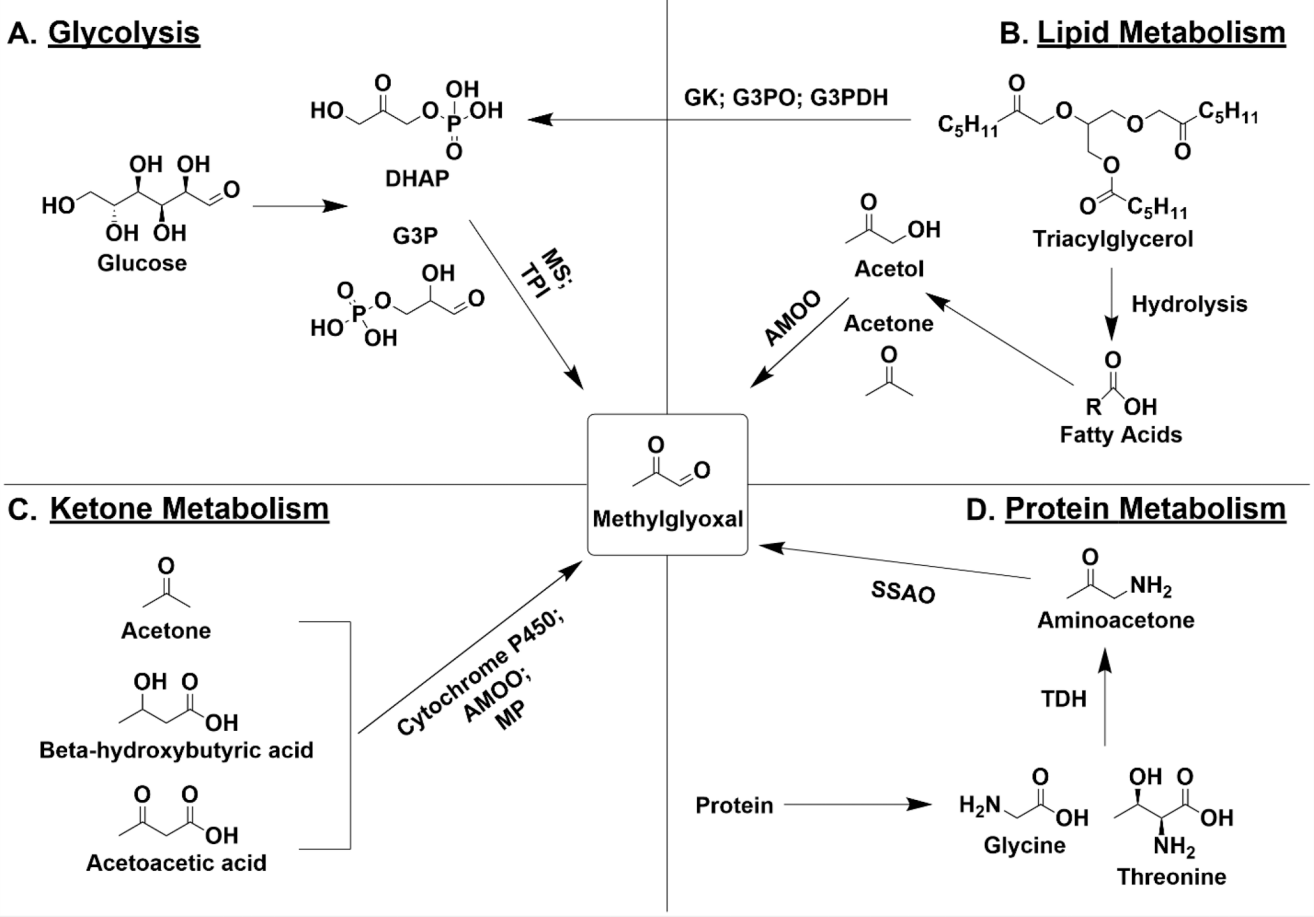
MG production from metabolic pathways. (A) During glycolysis, glucose is converted into DHAP or G3P which is metabolized by MS or TPI into MG. (B) In lipid metabolism, triacylglycerol undergoes hydrolysis to form fatty acids which are converted into acetol and acetone and then into MG by AMOO. (C) Ketones such as acetone, β-hydroxybutyric acid, and acetoacetic acid are converted to MG by cytochrome P450, AMOO, or MP. (D) During protein metabolism, amino acids such as glycine or threonine are metabolized by TDH into aminoacetone which is converted into MG by SSAO.

MG (2-oxopropanal or pyruvaldehyde) was discovered in the late 19th century as a byproduct of glucose, protein, and lipid metabolism.^9−12^ MG is proposed to exert its cellular effect through the formation of MG-AGEs on nucleic acids and protein, leading to changes in macromolecular stability and function.^10,13−15^ MG is predominantly produced as a byproduct of glycolysis during degradation of the triose phosphate intermediates, dihydroxyacetone phosphate (DHAP) and glyceraldehyde-3-phosphate (G3P) (Figure 1A).^16^ This occurs through two mechanisms: (1) nonenzymatic breakdown of G3P and DHAP, causing loss of the α-carbonyl group and phosphate, or (2) enzymatic conversion of G3P and DHAP into MG, mediated by enzymes such as MG synthase (MS) and triose phosphate isomerase (TPI) (Figure 1A).^16−18^ Sources of DHAP include the conversion of glucose to fructose and fructose 1,6-bisphosphate via sorbitol dehydrogenase and phosphofructokinase, respectively, which are then converted to DHAP via aldolase B.^19^ DHAP is also formed from metabolism of triacylglycerol into glycerol-3-phosphate via glycerol kinase (GK),^20^ followed by l-glycerol-3-phosphate oxidase (G3PO) or glycerol-3-phosphate dehydrogenase (G3PDH) (Figure 1A).^21,22^

MG is also produced from lipid, ketone, and protein metabolism.^19,23,24^ Triacylglycerol is hydrolyzed to fatty acids, which form acetol and acetone, giving rise to MG through acetol mono-oxygenase (AMOO) (Figure 1B).^25^ Ketone metabolism also produces MG through cytochrome P450, AMOO, and myeloperoxidase (MP) (Figure 1C).^26,27^ Finally, MG is formed from glycine and threonine metabolism to aminoacetone through threonine dehydrogenase (TDH). Aminoacetone is then converted to MG through semicarbazide-sensitive amine oxidase (SSAO) (Figure 1D).^28,29^

Collectively, this demonstrates that MG and its precursors and cofactors are abundant molecules associated with several metabolic pathways. It is important to note that MG abundance and its rate of formation also largely depends on the state of metabolic flux, the specific organism or tissue being studied, as well as physiological milieu. However, it has been estimated that intracellular MG levels range from 1 to 4 μM.^1^ However, due to MG’s reactive nature, it has been postulated that MG’s biological half-life is relatively short, and it is therefore likely that the actual amount produced is higher than current estimates.^30^ As a small molecule, MG is cell permeant and thus is able to diffuse through cell membranes from the extracellular space.^31^

## MG Regulation

Given that MG is reactive and can have detrimental impacts on cellular function, there are multiple mechanisms by which it is detoxified.

### MG Detoxification via the Glyoxalase System

**Figure 2:**
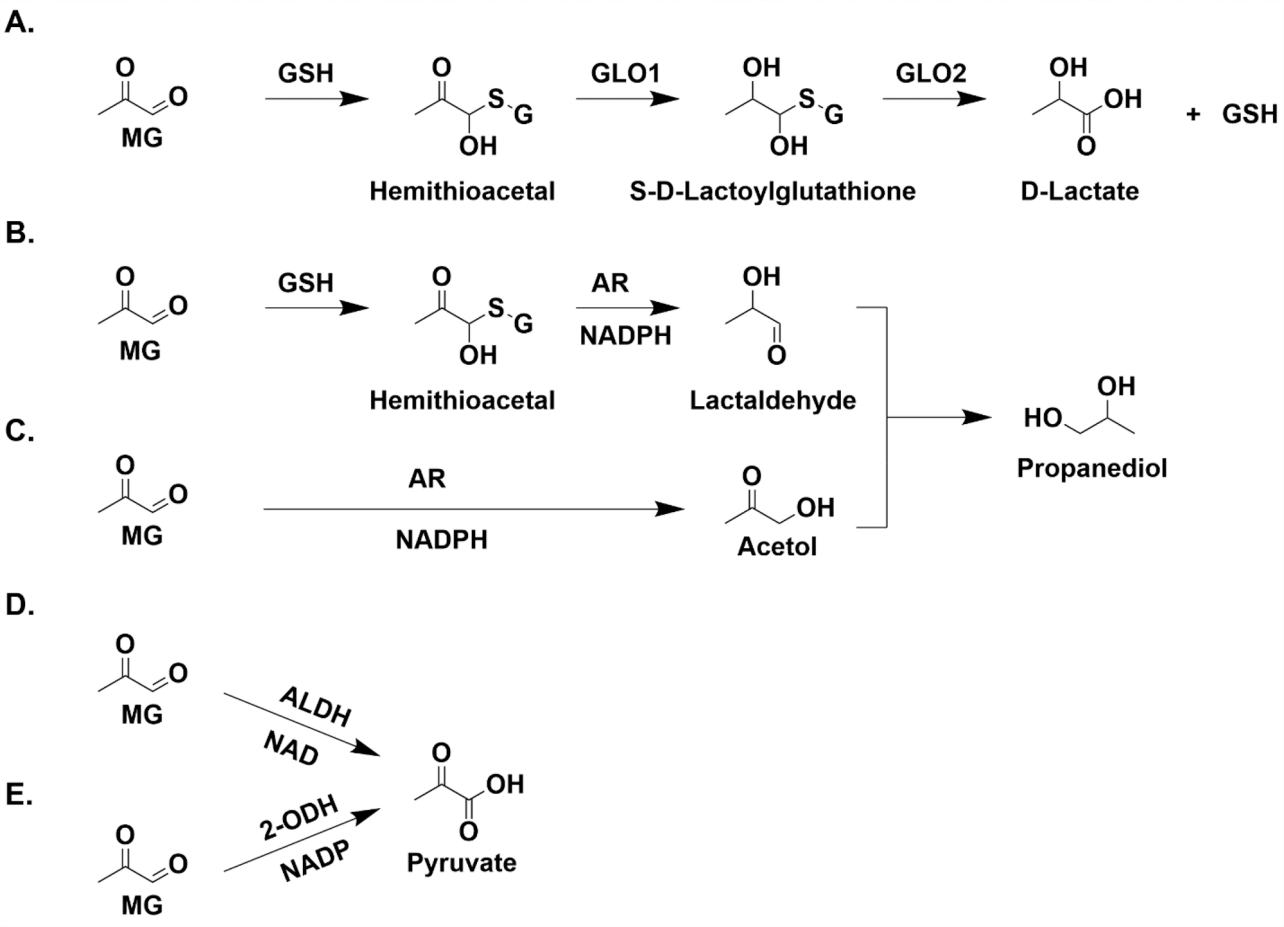
Various pathways for MG detoxification. (A) Glyoxalase system. MG reacts nonenzymatically with GSH to form a hemithioacetal which is recognized by GLO1. GLO1 converts the hemithioacetal to *S*-d-lactoylglutathione which is recognized by GLO2 to form d-lactate. (B) In addition to GLO1, AR also recognizes the hemithioacetal formed from the reaction of MG with GSH to lactaldehyde through an NADPH-dependent reaction. Lactaldehyde can undergo reduction, forming propanediol. (C) AR also recognizes MG directly through a GSH-independent mechanism, forming acetol which is reduced to form propanediol. GSH-independent reduction of MG by AR. (D) MG is oxidized by ALDH in an NAD-dependent mechanism to form pyruvate. (E) MG is oxidized by 2-ODH in an NADP-dependent reaction to form pyruvate.

One of the most prominent ways cells detoxify MG is through the glyoxalase pathway, a highly evolutionarily conserved system that involves the activity of two enzymes: glyoxalase 1 (GLO1) and glyoxalase 2 (GLO2).^32^ MG reacts nonenzymatically with glutathione (GSH) to form a hemithioacetal, which is recognized by GLO1 and converted into *S*-d-lactoylglutathione (Figure 2A).^33^ GLO2 then converts *S*-d-lactoylglutathione into d-lactate, regenerating GSH (Figure 2A).^33^ The rate-limiting step of this pathway is GLO1 recognition of the hemithioacetal.^34^ Recent work demonstrated a novel role for *S*-d-lactoylglutathione as a source of protein post-translational modifications, a process inhibited by GLO2.^35^

GLO1 overexpression protects cells from the accumulation of reactive oxygen species (ROS), glucose-driven apoptosis, and dysfunction arising from angiogenesis and diabetes.^36−39^ Recently, Alkb homologue 7 (ALKBH7) has been proposed to regulate GLO1 as *Alkbh7–/–* mice have elevated Glo1 expression and MG-protein adducts.^40^ In addition, DJ-1, also known as PARK7, is a gene implicated in Parkinson’s disease that detoxifies MG through a GSH-independent glyoxalase mechanism, converting MG to lactic acid.^41,42^ Clinical trials involving obese patients have implicated combinatory treatment of hesperetin and *trans*-resveratrol in inducing GLO1 expression and activity, which was found to lower their glucose levels and improve overall vascular function.^43^

A recent study was among the first to successfully create a viable GLO1 knockout mouse without significant detrimental side effects.^44,45^ However, the loss of GLO1 has not been fully characterized in mammalian models. A putative *Glo1* knockout mouse model was revealed to maintain a normal, healthy phenotype owing to gene duplication prior to gene trapping, preserving the wild-type phenotype.^46^ It was subsequently reported that aldehyde dehydrogenases (ALDH) and aldose reductases (AR), reviewed below, compensated for and mitigated the loss of GLO1.^44^ A similar finding implicating compensatory mechanisms for GLO1 has been reported in studies of GLO1 knockout zebrafish (*D. rerio*), which upregulated ALDH activity, partially compensating for the loss of GLO1.^47^ The loss of GLO1 in yeast (*S. cerevisiae*) resulted in hypersensitivity to MG and decreased cell survival and proliferation.^48,49^ In addition, the loss of GLO1 in fruit flies (*D. melanogaster*) led to obesity and prolonged lifespan and appeared to recapitulate some diabetic phenotypes, including hyperglycemia.^50^ In vitro, a *GLO1* knockout in HEK293T increased MG-AGEs, specifically MG-hydroimidazolone (MG-H1).^51^ Further studies in primary human aortic endothelial cells revealed that *GLO1* knockdown increased MG levels and subsequent inflammation, apoptosis, and dysfunction that led to vascular damage and impaired function.^52^ Taken together, this highlights the importance of the glyoxalase system as an indispensable mechanism for detoxifying MG.

### MG Detoxification via Oxidation

ALDHs are a class of nicotinamide adenine dinucleotide (NAD) and NAD phosphate (NADP)-dependent enzymes that oxidizes aldehydes to form carboxylic acids.^53^ ALDHs help detoxify aldehydes, a process that, if left unregulated, can be detrimental. For example, single-nucleotide polymorphisms, particularly ALDH2 rs672 G>A, and ALDH mutations are associated with an enhanced risk of heart disease,^54^ muscular dystrophy,^55^ and Alzheimer’s disease.^56,57^

The E1, E2, and E3 isozymes of ALDH react with MG and oxidize it into pyruvate in an NAD-dependent manner (Figure 2D).^58^ Likewise, 2-oxoaldehyde dehydrogenase (2-ODH) converts MG to pyruvate but in an NADP-dependent manner (Figure 2E).^59^ The loss of *Aldh* in murine models enhanced aldehydic adduct formation, cardiovascular and motor dysfunction, and tissue damage.^60^*Aldh* overexpression mitigated the effects of oxidative stress and ROS in various organs, both of which are upregulated following MG accumulation.^60,61^ Similar to *GLO1* knockout cells, *glo1* knockout in zebrafish moderately increased MG levels and significantly heightened Aldh activity, supporting the role of ALDH as an additional compensatory mechanism in the event the glyoxalase system is impaired.^62^

### MG Detoxification via Reduction

Aldose reductase (AR) is a 36 kDa enzyme encoded by the human *ALR2* gene and is part of the aldo-keto reductase enzyme family. The canonical role of AR is to reduce aldehydes into their respective sugar alcohols via the polyol pathway.^63^ AR activity is dependent on NADPH and exhibits a higher substrate selectivity and preference than ALDH, particularly for MG, thus making it more efficient at MG breakdown than ALDH.^64,65^ AR is associated with the development of diabetic complications, such as cardiovascular and renal diseases (reviewed in ref (65)). In addition, AR gene polymorphisms are associated with the risk of developing diabetic complications such as retinopathy,^66^ nephropathy,^67,68^ and neuropathy.^69^ For example, a CA dinucleotide polymorphism located in the 5′ promoter region of the *ALR2* gene is correlated with diabetic retinopathy.^66^ A similar biallelic polymorphism (C-106T) also in the promoter region of *ALR2* increased the risk of nephropathy, which was further enhanced if an individual carried both risk alleles.^49^ Patients with diabetic neuropathy have significantly lower frequency of the Z+2 allele than healthy controls.^69^ Therefore, AR polymorphisms appear to be closely related to the development of diabetic complications. AR-mediated MG detoxification operates in two distinct pathways: (1) GSH dependent in which the hemithioacetal formed between the nonenzymatic reaction with GSH and MG is converted by AR and NADPH to a lactaldehyde (Figure 2B) and (2) GSH independent in which MG reacts with AR and NADPH to form acetol (Figure 2C).^70^ Further AR-mediated metabolism of the lactaldehyde and acetol forms propanediol (Figure 2B and 2C). It is important to note that MG reduction by AR is significantly increased in the presence of GSH.^70^ In Schwann cells with *GLO1* knockout, AR inhibition increased intracellular MG levels and elevated sensitivity to MG.^71^ This suggests that not only is AR-mediated detoxification of MG important for MG elimination, both in the presence and in the absence of GSH, but also it is a compensatory mechanism if the glyoxalase system is impaired.^71^ However, the effects of AR overexpression have not been fully elucidated in the context of MG detoxification. AR overexpression was found to contribute to drug resistance in cancers,^72^ neural atrophy,^73^ and inflammation,^74,75^ contributing to a slew of disorders such as those impacting the eye^73^ and nerves.^76^ Given its ubiquitous nature, pursuing AR overexpression to promote MG breakdown may not be clinically apt.

## MG-AGEs on DNA and Protein

The electrophilic properties of MG drive its reaction with nucleophiles within macromolecules, forming covalent adducts. These adducts have been described for DNA and protein, and extensive work has been performed to determine the impact of these modifications on macromolecular function. Here, we will discuss the main adducts formed on DNA and protein, the pathways cells use to remove these adducts, and the impact of adducts on macromolecular function.^77−79^

## MG-AGEs on Nucleic Acids: Impact on Structure and Repair

### MG-Nucleic Acid Adducts

**Figure 3:**
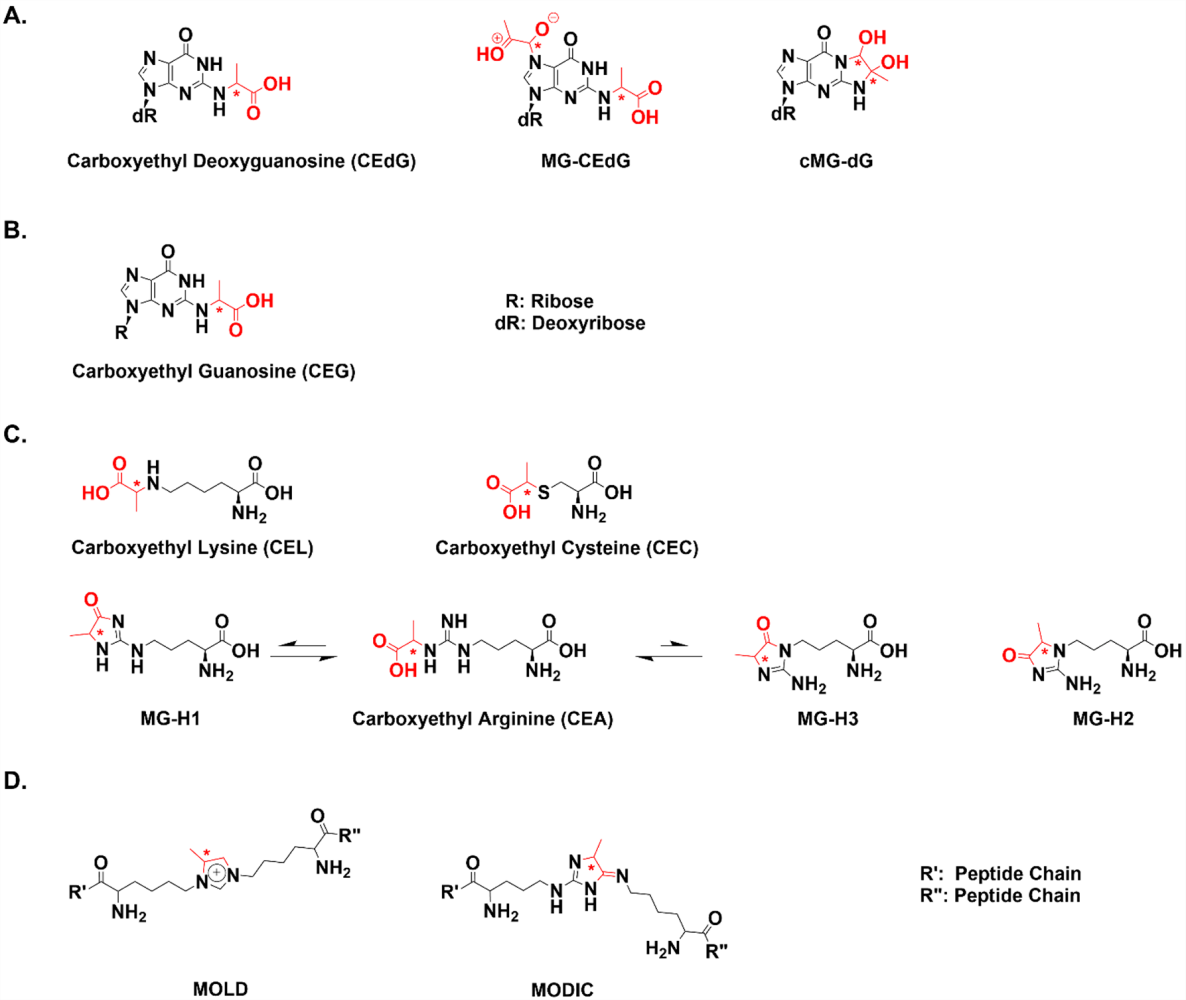
Chemical structures of MG-modified nucleic and amino acids. (A) MG modifies dG, forming three main adducts, CEdG, cMG-dG, and MG-CEdG. Stereocenters are indicated by asterisks, and MG addition is shown in red. dR represents the deoxyribose sugar. (B) Proposed structure of MG-modified RNA adduct CEG. R represents the ribose sugar. (C) Arginine, lysine, and cysteine are primary targets for MG modification. Lysine modification forms CEL, and arginine modification forms MG-H1, MG-H2, and MG-H3. MG-H1 and MG-H3 can be hydrolyzed to form CEA. (D) MG can form lysine dimers MOLD and MODIC.

The primary target for MG modification in DNA is deoxyguanosine (dG).^79^ A 20-fold excess of MG with dG resulted in the formation of a cyclic dihydroimidazolone 1,*N*^2^-(1,2-dihydroxy-2-methyl)ethano-dG (cMG-dG) (Figure 3A).^7^ Additional adducts were later characterized including a product with 2-MG equivalents, *N*^2^-(1-carboxyethyl)-7-1-hydroxy-2-oxopropyl-dG (MG-CEdG) and *N*^2^-(1-carboxyethyl)-2′-dG (CEdG), which was formed at less than stoichiometric amounts of MG (Figure 3A).^13,14,80^ Although these adducts are formed in vitro, CEdG is the only adduct observed in genomic DNA.^13^ This is proposed to occur because cMG-dG is unstable and degrades to generate hydrated MG and dG. This hydrated form of MG only modifies dG at the *N*^2^ position, driving CEdG production. cMG-dG does not directly convert to CEdG, as was previously hypothesized.^13^ In addition to DNA, we hypothesize that MG modifies guanosine nucleotides in RNA to form *N*^2^-(1-carboxyethyl)-guanosine (CEG) (Figure 3B).^81^

### Regulation of MG Adducts—DNA Repair

DNA adducts induce genomic instability, impact transcription, and are mutagenic. To prevent this, cells have multiple DNA repair pathways, including nucleotide excision repair (NER), base excision repair (BER), and mismatch repair (MMR). Each pathway varies in damage recognition and removal.^82^ The repair pathway activated depends on the chemistry of the lesion and how it perturbs the DNA structure; each repair pathway employs different proteins that have specific interactions with the DNA to trigger the repair pathway. For instance, the NER pathway is the primary pathway cells use to repair bulky DNA adducts that induce helical distortions,^83^ while BER repairs small lesions that do not significantly distort the helix,^84^ and MMR removes mispaired bases in the genome.^85^ The primary pathway for the removal of MG-DNA adducts is not clear, but both BER and NER are proposed to play a role because shuttle vectors modified with MG have persistent DNA adducts when replicated in cells deficient in XPG, a protein involved in both BER and NER.^86^

DNA repair efficiency is impacted by protein expression and activity and is regulated by complex mechanisms. Hyperglycemia, which increases the levels of MG-DNA adducts, may also play a role in regulating DNA repair. Ciminera et al. recently showed that high glucose decreased the expression of proteins in the NER pathway, leading to accumulation of MG-DNA adducts corresponding to decreased functional repair.^87^ The authors suggested that high glucose may downregulate NER protein expression through a HIF-1α-dependent mechanism.^87^

**Figure 4:**
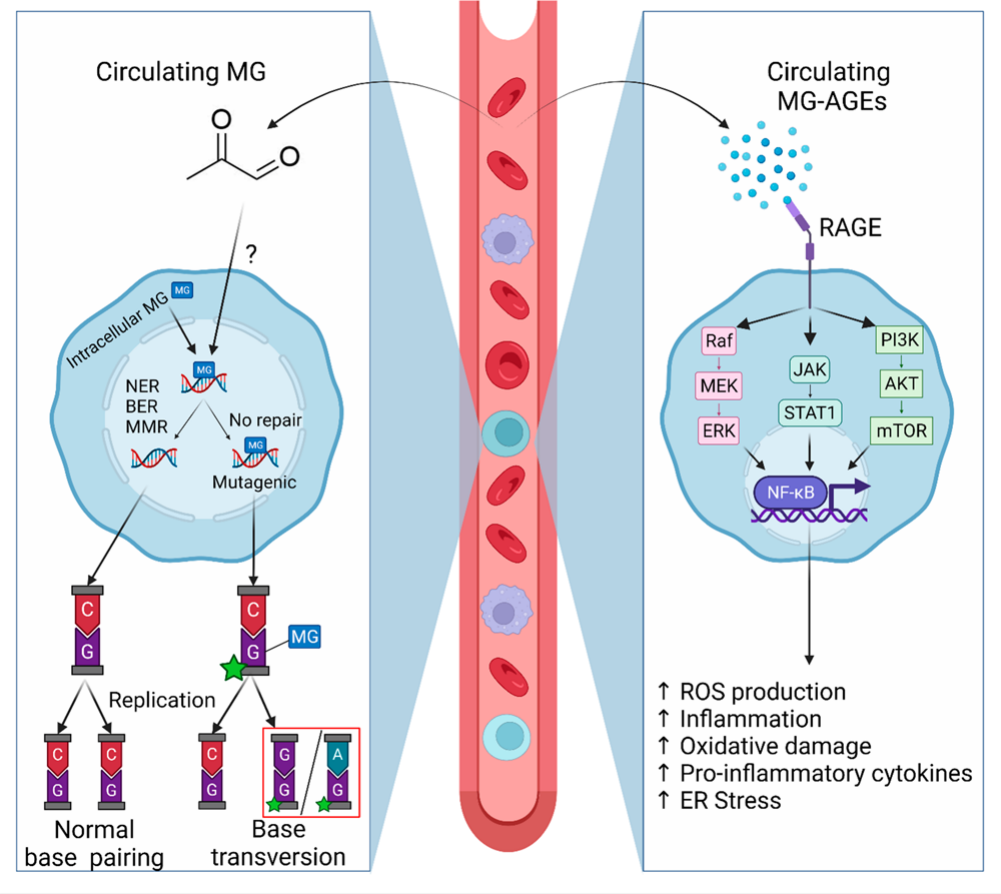
MG forms DNA MG-AGEs. (A) Adducts formed on deoxyguanosine bases can lead to improper base transversions in which guanine incorrectly base pairs with either adenine or guanine. (B) AGEs activate RAGE, and downstream signaling cascades to trigger NF-kB activation, leading to ROS production, inflammation, oxidative stress, etc. Created with BioRender.com.

When cells are unable to efficiently repair DNA damage, it leads to persistent lesions that may cause genomic instability and mutations (Figure 4A). CEdG is associated with single-strand DNA breaks and increased mutation frequency.^88^ In *S. cerevisiae* D7, MG induced both mitotic gene conversion and reverse point mutations with a dose-dependent response in mutation frequency.^89^ Forward selection analysis for mutations in the hypoxanthine phosphoribosyl transferase (HPRT) gene also revealed MG to be a mutagen in Chinese hamster lung cells and T-cell lymphocytes.^90,91^ Mutagenesis in T cells was observed with both a single high-dose MG treatment (1 mM) and multiple low-dose MG treatments (0.1 mM).^91^

To define the specific mutations induced by MG, Tamae et al. utilized MG-modified shuttle vectors.^86^ The MG-adduct density was quantified, and the shuttle vectors were transfected into XPG-proficient or -deficient human fibroblasts, a protein involved in both BER and NER.^92^ A linear, dose-dependent increase in mutations was observed in the *supF* tRNA marker gene.^86^ However, there was a maximal elevated mutation frequency in the XPG-deficient fibroblast cells at varying adduct densities up to 5-fold background levels.^86^ Specific sites in the *supF* gene were preferentially modified, suggesting that there may be sites more susceptible to MG modification. These results were recapitulated by Murata-Kamiya et al., who showed that MG-modified shuttle vectors had guanine transversions when replicated in COS-7 fibroblast cells.^93^ Further characterization of these mutations via sequencing analysis revealed that multibase deletions and base-pair substitutions were predominant in the mutant signature with the latter being primarily G:C → C:G and G:C → T:A transversions in the *supF* gene of the shuttle vector (Figure 4A).^85^*E. coli* deficient in NER also showed increased levels of MG-induced mutations.^94^ In addition, treatment of human melanoma WM-2664 cells with MG induced the formation of CEdG adducts, which had mutagenic properties in *E. coli* and were a substrate for DinB DNA polymerase, a known contributor to mutagenesis.^95^

## MG-Protein Adducts: Formation and Impact on Structure and Function

### MG-Protein Adduct Formation

In addition to DNA, MG modifies amino acids including lysine, arginine, and cysteine, forming protein adducts that can impact the structure and function (Figure 3C). Initially, Takahashi found that high MG concentrations modified free amine-containing amino acids, specifically lysine and arginine, and hypothesized that thiol-containing amino acids could be modified as well.^96^ This was later confirmed by Lo et al., who demonstrated that MG modifies cysteine, forming a reversible hemithioacetal, later named carboxyethyl cysteine (CEC).^78^ Physiological MG concentrations modify proteins, particularly BSA, producing the fluorescent imidazole derivative MG-H1 (Nδ-(5-hydro-5-methyl-4-imidazolon-2-yl)ornithine).^77^ MG-H1 is the predominant adduct, but two other hydroimidazolones also form: MG-H2 (2-amino-5-(2-amino-5-hydro-5-methyl-4-imidazolon-1-yl)pentanoic acid) and the significantly less abundant MG-H3 (2-amino-5-(2-amino-4-hydro-4-methyl-5-imidazolon-1-yl)pentanoic acid).^77^ MG-H3 can be hydrolyzed to form carboxyethyl arginine (CEA).^97^ It was previously hypothesized that MG-H1 is resistant to hydrolysis, but McEwen et al. recently demonstrated that it is also hydrolyzed to form CEA.^98^ MG also modifies Nα-acetyllysine, forming a glycosylamine, N^ε^-carboxyethyllysine (CEL) (Figure 3C).^78^ CEL is found in lens protein and is associated with age.^99^ While there is significant CEL formation in vitro, MG-H1 is the most abundant amino acid adduct in clinical samples, approximately 10 times greater than CEL.^100^

Adducted amino acids can undergo a secondary modification, resulting in macromolecule cross-linking. MG forms the protein dimers lysine–lysine (MOLD) and lysine–arginine (MODIC) (Figure 3D).^101^ Cross-linking also occurs between DNA and polymerases, which is proposed to occur through a dG–lysine bond.^102,103^

### Impact of MG-AGEs on Protein Structure and Function

MG modification of amino acids disrupts the enzymatic activity and protein structure.^104,105^ Endothelial cells exposed to hyperglycemia had increased cellular MG concentrations and protein MG-AGEs.^106^ Proteomics analysis revealed that 17% of proteins had low-level MG modification including those involved in protein synthesis, protein folding, kinase signaling, glycolysis, and gluconeogenesis.^106^ The proteins with the highest number of modifications were pyruvate kinase M, β-actin, α-enolase, and heat shock protein (HSP) 90-β.^106^ HSPs are chaperone proteins that are elevated in response to stress, and in addition to HSP90-β, MG also modifies HSP6, HSP27, and α-crystallin (protein with HSP domains). This interrupts their interactions with other proteins and impairs chaperone function.^107−110^ Collagen modified with MG shows a decreased ability to adhere to mesangial cells.^111^ Human serum albumin is also a target for MG modification.^112^ There is a hot spot of modification at Arg410, which is located in drug binding site II and in the active site for esterase activity.^112^ HIF-1α modification by MG decreases heterodimer formation and promoter binding.^113^ This is a potential mechanism for the decreased DNA repair protein expression in cells grown in high glucose observed by Ciminera et al. as DNA repair proteins are targets for HIF-1α transcription activity.^87^ Recently, work from Marnett demonstrated that histones are targets for MG modification, potentially regulating gene expression.^51^ Histone modification by MG has also been associated with changes in chromatin architecture related to disease.^114^

## RAGE Activation and Signaling by MG-AGEs

In addition to changing DNA and protein expression and stability, MG-AGEs are proposed to impact cell function by binding to and activating the receptor for AGEs (RAGE). RAGE is an immunoglobulin transmembrane pattern-recognition receptor that is expressed on a range of cells, including endothelial,^115,116^ immune,^116,117^ skeletal muscle,^118^ and cancer cells.^119−121^ RAGE is generally present in three primary forms, as a full-length membrane-bound receptor (flRAGE), as a soluble product (sRAGE) created by ADAM10-mediated cleavage of flRAGE, and as a splice variant known as endogenous secretory RAGE (esRAGE).^122^ Binding of MG-AGEs to RAGE has been investigated both with free modified amino acids and with modified proteins such as albumin. Xie et al. found that CEL does not bind to RAGE, but protein with CEL modifications does.^123^ However, nuclear magnetic resonance (NMR) studies revealed that CEL-containing peptides bind specifically to a positively charged moiety of the V domain of RAGE.^124^ Additional NMR studies demonstrated that MG-derived hydroimidazolones (MG-H1–3) bind to a positively charged pocket of the V domain of RAGE, similar to CEL.^125^ MG-modified albumin (MG-BSA) also binds to RAGE to cause signal transduction; treatment of A549 adenocarcinoma cells with MG-BSA caused an upregulation of JNK phosphorylation in a RAGE-dependent mechanism.^125^

In addition to AGEs, RAGE is bound by structurally diverse ligands, including phosphatidylserine,^126^ high-mobility group box-1 (HMGB1) protein,^127^ S100b,^128^ lipopolysaccharides,^126,129^ and nucleic acids.^130^ RAGE activates JAK/STAT,^131^ PI3K/AKT/mTOR,^132^ and MAPK/ERK,^133^ leading to NFκB activation (Figure 4B).^134^ This upregulates ER stress, ROS production, inflammation, and oxidative damage (Figure 4B).^135,136^ The NFκB cascade plays a significant role in mediating cellular responses including inflammation, apoptosis, and cellular survival and proliferation.^137^ Therefore, AGE-dependent activation of the NFκB pathway via RAGE contributes to numerous disease pathologies.^131,138,139^

The signaling cascade triggered by RAGE activation is influenced by cell type. For instance, RAGE activation can be detrimental to normal cell growth but advantageous for malignant cell growth. In cancer, increased RAGE expression is correlated with a worse clinical prognosis, which is supported by AGE/RAGE signaling, driving survival,^140^ proliferation,^140^ migration,^141^ angiogenesis,^142^ and metastasis.^142^ Therefore, AGE/RAGE signaling is proposed to be a therapeutic target to prevent cancer onset and progression.

## Physiological Impact of MG and MG-AGEs

### MG and MG-AGEs in Disease

**Figure 5:**
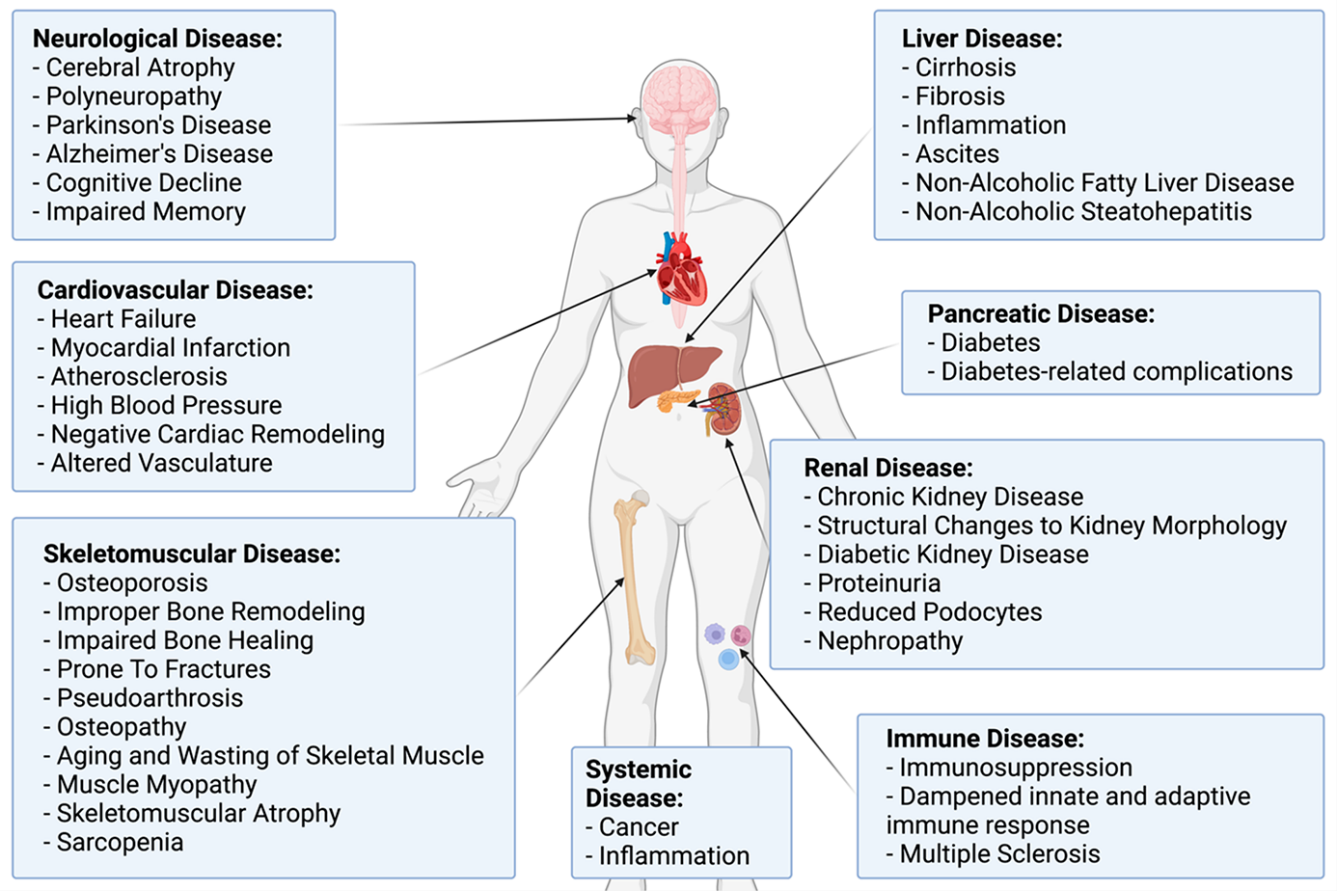
MG and MG-AGEs have systemic physiological impacts on the body. MG and MG-AGEs have been implicated in the pathogenesis of numerous diseases throughout the body. This includes the brain, heart, skeletomuscular system, liver, pancreas, kidney, and immune system. Created with BioRender.com.

MG and MG-AGEs are associated with the pathogenesis of numerous diseases including cancer, diabetes, and cardiac disease.^143−146^ Here, we discuss the clinical relevance of MG and MG-AGEs in different human diseases and the impact of MG-AGEs in the context of RAGE (Figure 5).

### MG and MG-AGEs in Cardiovascular Disease

Patients with heart failure secondary to diabetes have increased MG-AGEs on actin and myosin in heart muscle when compared to nonfailing controls or heart failure in individuals without diabetes.^147^ These modifications interfere with protein interaction with muscle and disrupt calcium sensitivity, both processes that are critical for proper cardiac function.^147^ In a study of comorbidity with HIV infection, a positive association between MG in the heart and plasma and an enhanced risk for heart failure was observed.^148^ Moreover, higher plasma MG levels were consistently associated with fatal cardiovascular events in individuals with type 1 diabetes (T1D).^149^ A similar finding was also made in individuals with type 2 diabetes (T2D) with elevated plasma MG associated with higher risk for atherosclerosis and high blood pressure, spurring interest in its utility as a predictive biomarker for heart disease.^150^

In mice, MG-AGEs in the heart were associated with worse outcomes following myocardial infarction, specifically negative cardiac remodeling and cardiac dysfunction.^151^ In hypertensive rats, elevated aortic and plasma levels of MG and MG-AGEs were associated with oxidative stress, endothelial dysfunction, high blood pressure, and altered vasculature.^152^ These findings have since been recapitulated in patients with diabetes; elevated plasma and serum MG-AGEs were associated with microvascular complications, higher blood pressure, and markers of atherosclerosis and coronary heart disease.^153−157^

The AGE/RAGE axis, which describes the interplay between AGE production, binding to RAGE, and RAGE activation, has been associated with coronary artery disease resulting from hyperglycemia due to insulin resistance from T2D.^158−160^ AGEs are proposed to lead to cardiac dysfunction by cross-linking low-density lipoproteins and extracellular matrix proteins such as collagen and elastin, causing stiffening of the blood vessel lining.^159^ Blockade of the AGE/RAGE axis via administration of sRAGE in mice demonstrated a dose-dependent drop in markers of atherosclerosis development.^161^ Taken together, this suggests that MG-AGEs may serve as an early indicator of impending atherosclerosis development and reduced heart function.

### MG and MG-AGEs in Neurological Disease

MG and MG-AGEs are associated with the development of neurological disorders such as Alzheimer’s disease (AD), cerebral atrophy, polyneuropathies, and Parkinson’s disease (PD).^162−165^ In humans, there is a negative association between serum MG levels and memory, overall executive function, and lower gray matter volume.^162,166^ This suggests that MG is associated with subsequent neurodegeneration and cognitive decline, particularly in older people. In AD, MG and MG-AGEs are posited to accumulate in β-amyloid plaque deposits and neurofibrillarytangles.^167,168^ In neuroblastoma cells, MG was neurotoxic and associated with increased ROS levels, triggering neuronal damage commonly seen in PD.^169^ In nerve biopsies of patients with vasculitic polyneuropathy, increased MG-AGEs, RAGE, and NFκB expression were detected in neural mononuclear cells and vessels.^170−173^

How MG and MG-AGEs drive cognitive decline and neurodegeneration is not known; however, they are proposed to trigger mitochondrial damage and inflammation in a RAGE-dependent manner.^174^ Older mice have elevated MG-AGEs in their cerebral cortices and hippocampi, leading to mitochondrial dysfunction.^175^ This observation was recapitulated in rats with streptozotocin-induced AD, which exhibited persistent activation of the MG/AGE/RAGE/NOX-2 pathway.^176^ Transgenic mouse models mimicking AD have increased expression and activation of RAGE in astrocytes, particularly those in the hippocampus, a key memory controller.^177^ The brains of patients with AD have nearly 2-fold higher MG levels than control individuals, with MG being 5–7 times higher in cerebrospinal fluid than in plasma.^178^ Likewise, AD patients have significantly higher hippocampi MG-AGEs, a finding recapitulated in the nigra neurons of PD patients.^179^ Furthermore, MG-AGEs are elevated in adipose tissue of patients with neuropathy, AD, or neural aging.^180−182^ MG and MG-AGEs accumulate in glia and astrocytes, which have an increased expression of RAGE positively correlated with age.^177,183−185^ This suggests a possible link between elevated MG levels, increased MG-AGE production and accumulation, and their dispersal via the cerebrospinal fluid system, driving RAGE activation on cells throughout the central nervous system and driving disease onset and progression.

### MG and MG-AGEs in Skeletomuscular Disease

Recent studies have highlighted the impact of MG and MG-AGEs on the skeletomuscular system.^186^ MG promotes bone degeneration; in both in vivo rat and in vitro models, MG led to osteoclastogenesis, a step in the osteoporosis development.^187,188^ In the macrophage cell line RAW264.7, MG activated c-Jun N-terminal kinases, suggesting MG propagates improper bone remodeling via the JNK pathway.^187^ Patients with diabetes are prone to developing pseudoarthrosis in which their ability to heal bone fractures is delayed.^189^ Diabetic mice exposed to MG and given a bone defect exhibited significantly delayed bone healing and osteoblast differentiation in a dose-dependent manner as opposed to nondiabetic control mice.^190^ Interestingly, they also had elevated levels of serum and bone MG-AGEs.^190^ This suggests that MG detoxification may mitigate bone degeneration and loss in patients with diabetes. Limonene, an antioxidative terpene, also mitigates the effects of MG on diabetic osteopathy; pretreatment of murine osteoblast cell line MC3T3-E1 with limonene reduced endoplasmic reticulum stress, ROS release, and cell death, which was recapitulated using spironolactone.^191,192^ However, the precise mechanism of this protective effect by both limonene and spironolactone is not fully elucidated.

Canonically, RAGE signaling in skeletal muscle is involved in normal skeletomuscular function and maintenance; however, depending on the ligand, RAGE activation also causes wasting, inflammation, and skeletal muscle aging.^143,193,194^ These effects are proposed to occur by AGE-mediated aging and cross-linking of critical components of the extracellular matrix such as collagen and the basal lamina.^195,196^ Collectively, this leaves the individual prone to developing overt skeletomuscular atrophy, sarcopenia, and osteoporosis.^193,197^ In myoblast cells, MG-AGEs increased oxidative stress and reduced myotube formation while upregulating RAGE expression and activation.^198^ In diabetic mice, skeletal muscle and plasma have significantly higher MG-AGEs compared to controls, suggesting a link between MG-AGE production and accumulation in the skeletal muscle.^198^ These findings were corroborated in vitro with MG treatment of C2C12 mouse myotube cells, resulting in higher levels of MG-AGEs.^198^ A mechanism for AGE/RAGE-mediated pathogenesis of myopathy occurred via AMPK-downregulation of the Akt cascade, exacerbating skeletomuscular dysfunction and subsequent loss.^199^

### MG and MG-AGEs in Renal Disease

Kidney disease is associated with hyperglycemia and metabolic dysfunction, suggesting an association with MG and MG-AGEs. Tezuka et al. conducted an observational study with 150 individuals at different stages of chronic kidney disease (CKD) with the goal of measuring plasma MG levels.^200^ They found plasma MG was positively associated with CKD, thus identifying MG as a potential tool for estimating kidney disease prognosis and stage.^200^ Rats treated with MG intragastrically had increased expression of mRNA of pro-inflammatory and oxidative pathways in the kidney transcriptome.^201^ This data was further supported by proteomic and metabolomic analyses which revealed heightened secretion of extracellular matrix components and membrane phospholipids in MG-treated rats, both of which are indispensable for proper kidney function.^201,202^ Furthermore, *Glo1*-deficient nondiabetic mice had altered kidney morphology akin to that of diabetic nephropathy, suggesting that MG contributes to the damage that is observed in CKD.^203^ Similarly, *Glo1* overexpression in diabetic mice and rats mitigated MG-AGE production and subsequent oxidative stress, diabetic kidney disease, and retinopathy.^203,204^ Protein cross-linking by AGEs contributes heavily to tissue damage and is a marker for organ dysfunction.^205^ Measurement of MG-AGEs has also been used to predict kidney disease prognosis and progression.^206−208^ Serum MG-AGEs are significantly, positively associated with decreased renal function in humans.^209^ Individuals with T1D showed higher urinary excretion of MG-AGEs that proved useful as an indicator of early renal failure.^210,211^ In individuals with end-stage CKD, MG-AGEs were strongly correlated with indicators of endothelial dysfunction and inflammation.^212,213^

Recently, Lee et al. made a similar finding in humans, demonstrating that in human mesangial cell lines, MG-AGEs trigger nephropathy via upregulating RAGE expression, leading to ROS production and activation of PI3K/AKT and NFκB.^214^ RAGE suppression via small-molecule inhibition and siRNA diminished oxidative stress and inflammatory response, suggesting the MG-AGE/RAGE axis contributes to nephrotic damage and dysfunction.^215^ This was also observed in rats with streptozotocin-induced nephropathy, in which treatment with Moutan Cortex mitigated AGE-induced inflammation, resulting in a protective effect.^216^ MG-AGEs also act through the RAGE/JNK pathway, causing mitochondrial and ER stress dysfunction as well as an upregulation of apoptotic markers such as p53 and Bax.^217^ Furthermore, gliclazide, a therapeutic for diabetes, conferred a protective effect on renal damage and dysfunction induced by MG-AGEs and hyperglycemia through inhibition of the AGE/RAGE/ROS/NFκB cascade.^218^ It is important to note that not every AGE/RAGE interaction triggers the same pathways; Baragetti et al. identified −374 T/A polymorphisms in RAGE that increased the risk of progression to CKD.^219^ This suggests that in renal dysfunction it may be beneficial to target downstream RAGE signaling, as opposed to upstream MG-AGE/RAGE binding.

### MG and MG-AGEs in Liver Disease

In rats given carbon tetrachloride (CCl_4_) to induce early-stage hepatitis, liver MG levels and d-lactate were elevated compared to control untreated rats.^220^ Similarly, a clinical trial conducted by Michel et al. revealed a positive correlation between elevated levels of serum MG, liver cirrhosis, and widespread inflammation.^221^ Elevated serum and circulating MG levels were correlated with worsening liver disease prognosis and increased risk of developing additional liver-related complications, such as ascites.^221^ In HepG2 cells, MG impaired mitochondria, caused cell death via apoptosis, promoted ROS production, and diminished GSH levels, a critical component of the glyoxalase detoxification system.^222^ In vivo, MG administration led to acute liver toxicity as evidenced by elevated levels of alanine aminotransferase and aspartate aminotransferase, both indicators of liver health. Taken together, this suggests that MG induces liver disease by triggering mitochondrial dysfunction and oxidative stress as a result of excess ROS production.^222^

To determine the impact of inflammation on MG regulation, rats were treated with CCl_4_, which led to MG accumulation in the liver, causing decreased Glo1 expression, increased production of MG-AGEs, and RAGE activation, causing inflammation and stress.^223^ The impact of MG on decreased Glo1 expression is intriguing as it suggests a positive feedback loop in which elevated MG prevents its own detoxification while continuing to cause hepatic dysfunction.^223^ Excess MG leads to further MG-AGE production, and several studies have identified elevated levels of MG-AGEs in both the plasma and the serum of patients with liver disease and in obese mice.^224−226^ Serum MG-AGEs and sRAGE in nondiabetic patients are associated with nonalcoholic fatty liver disease.^227^ In addition, liver steatosis and inflammation led to elevated levels of circulating MG-AGEs.^228^

Activation of the MG-AGE/RAGE axis increased apoptosis and TGF-β, TNF-α, IL-8, and IFN-γ levels.^229,230^ Further characterization of the crosstalk between the pro- and the anti-inflammatory cytokines released as a result of the AGE/RAGE axis in liver disease is needed to understand their role in hepatic dysfunction. The upregulation of these cytokines appears to be ablated upon administration of a siRNA-targeting RAGE in primary rat hepatic stellate cells and prevented overt disease progression.^231^ Subsequent in vivo administration of RAGE siRNA in rats recapitulated these in vitro findings and delayed the development of liver fibrosis via hampered activation of NFκB.^232^ Interestingly, deletion of *Ager* (the gene that encodes RAGE) did not prevent development of liver steatosis, which suggests an alternate RAGE-independent mechanism of liver dysfunction mediated by MG-AGEs.^226,233^

### MG and MG-AGEs in Immune Disorders

Recent studies have implicated MG as a potent immunosuppressor. Price et al. were among the first to show that increased MG inhibited T-cell proliferation and triggered a loss of both pro- and anti-inflammatory cytokines, such as IFN-γ in myeloid cells and TNF-α and IL-10 in T cells.^234^ MG also reduced metabolic activity in myeloid-derived suppressor cells, a group of regulatory immune cells of myeloid origin.^235^ This suppressive phenotype was also found in CD8+ cytotoxic T cells, which was proposed to occur via MG transfer, thus causing further immunosuppression.^235^ MG modification of histone H2A increased immunogenicity, providing some evidence that it may be involved in the autoimmune response in cancer and generation of autoantibodies.^236^

In vitro studies on the effects of MG-AGE accumulation on immune cells revealed that MG-AGEs impaired the activation of inflammasomes in macrophages, thus hampering innate immunosurveillance.^237^ This effect was independent of MG-AGEs binding to RAGE but rather occurred through the suppression of macrophage M1 polarization, which would otherwise trigger a pro-inflammatory state prone to phagocytosing foreign or unwanted material.^237^ Jin et al. found conflicting results, noting that MG-AGEs elevated RAGE expression in macrophages, subsequently triggering macrophage polarization into a pro-inflammatory M1 phenotype through NFκB pathway activation.^238^

RAGE is expressed on T cells and antigen-presenting cells such as macrophages, dendritic cells, and B cells, and its activation drives both innate and adaptive immune responses.^239^ For example, the RAGE cascade is critical for T-cell priming and proliferation and mediates interactions between dendritic cells and T cells during cross-priming to propagate an adaptive immune response.^240,241^ However, this can have adverse effects in disease. RAGE is present on macrophages and microglia, which are two major cell groups that infiltrate and attack the central nervous system, leading to multiple sclerosis. In vivo clinical studies in patients with multiple sclerosis demonstrated an increase in MG and MG-AGEs in astrocytes and cerebrospinal fluid.^183^ This suggests that circulating MG-AGEs may have a role in paracrine signaling in the body by traveling to and activating RAGE-expressing immune cells, thus exacerbating the immune attack on the nerves.^183,242^ In multiple sclerosis patients, plasma CEL levels were significantly higher than their control counterparts and were also correlated with rate of relapse.^243^

### MG and MG-AGEs in Cancer

Current literature suggests that MG has a hormetic effect in cancer, serving a pro-tumorigenic role in certain conditions and an antitumorigenic effect in others. A selection of studies investigating the pro- or antitumor role of MG and MG-AGEs in various cancers is summarized in Table 1. As reviewed by Leone et al., there is an inverse correlation between MG concentration and cancer growth and metastasis.^144^ Cancers preferentially use glycolysis and have enhanced glucose uptake; they produce higher levels of MG.^244^ To counteract this, cancers overexpress GLO1, which may lead to oversaturation of available GLO1, leading to MG accumulation and toxicity.^245−249^ Nokin et al. demonstrated that preconditioning cells with high glucose (500 mM) or MG (2.5 mM) successfully conditioned yeast to be more tolerant and resistant to higher concentrations of MG (20 mM) and subsequent oxidative damage, an effect found to be independent of GLO1.^250^ This is posited to be due to a hormetic mechanism by MG in that at low levels MG drives cancer growth but can cause adverse effects at high levels.^250^

**Table 1:** Effects of MG and/or MG-AGEs on Cancer

cancer type	MG/MG-AGE	pro/anti cancer	model	dose	effect	mechanism	source
breast	MG	pro	MDA-MB-231, MDA-MB-468, MCF7 cells	300 μM	increase growth and metastatic potential	increase Hsp90 glycation, carbonyl stress, and YAP and TAZ accumulation	(291)
	MG	pro	MDA-MB-231, MDA-MB-468, MCF7 cells	300–500 μM	increase metastasis and migration	activation of MEK/ERK/SMAD1 pathway; promotes ECM remodeling	(292)
	MG	anti	MCF7, T47D, MDA-MB-231 cells	100–800 μM	decrease viability, colony formation, migration, and invasion; increase apoptosis	increase p-MAPK, p-JNK, and p-ERK; decrease Bcl-2 expression	(293)
	MG-AGE	pro	MDA-MB-231 cells	25–100 μg/mL	increase proliferation, invasion, and migration	upregulated MMP9 and RAGE; p-ERK and p-p70S6K1	(294)
	MG-AGE	pro	primary human TNBC samples		increase tumor aggression and progression	increase MG detoxification; protected against dicarbonyl stress	(295)
	MG and MG-GE	pro	MCF7 cells	25–200 μg/mL	increase viability, proliferation, migration	AGE-mediated activation of RAGE and p-ERK; increase CREBP	(296)
brain	MG	anti	T98G and U87MG cells	25 μM	changes in cell cycle, inhibited proliferation; increase apoptosis, senescence	cells arrest in G1/G0; proliferation inhibited	(297)
kidney	MG-AGE	pro	786-O, A498, and HK-2 cells	100–800 μg/mL	increase proliferation, survival, migration; decrease apoptosis	increase PCNA, MMPs p-AKT, pERK; decrease Bax and Caspase 3	(298)
liver	MG	anti	Huh-7, HepG2, Hep3B cells	1 μM	decrease migration, invasion, adhesion	proposed to be p53 dependent	(299−301)
leukemia	MG	anti	HL-60 cells	0–1 mM, 0–524 μM	decrease viability/proliferation; increase DNA damage/apoptosis	cells arrested in G1 with nuclear DNA fragments similar to apoptosis	(291,292)
prostate	MG	anti	PC-3 cells	0–5 mM	decrease growth, increase apoptosis	downregulated cyclin expression and degraded PARP-induced G1 arrest, blocked glycolysis	(302)
thyroid	MG and MG-AGE	pro	patient samples; B-CPAP, TPC1, 8505C, CAL62 cells	5 μM	increase cancer aggression, lethality, invasion/migration	differential E-cad, vimentin, MMP-1, TGF-β1 expression, and increase FAK signaling pathway	(303)
colon	MG	pro	CT26 mouse models	50 mg/kg	increase proliferation, migration, inflammation, oxidative stress	increase IL-6 secretion, increase p-ERK, p-p38 MAPK, p-PI3K, and p-mTOR	(304)
	MG	anti	SW480, SW620, DLD-1, HCT15 cells; CRC mouse models	400–1600 μM	decrease growth, proliferation, migration, colony formation; increase apoptosis	increase STAT1, p53, Bax; decrease c-Myc and Bcl-2	(305)
	MG	anti	DLD-1 and SW480 cells; CRC mouse models	25–2000 μM	decrease viability, migration, invasion, proliferation, growth; increase apoptosis	decrease c-Myc; interfered with glycolysis (less ATP and lactate made, less glucose used)	(306)
	MG-AGE	pro	primary human samples		EMT progression and increase tumor aggressiveness; cytokine immunomodulation to promote tumor growth	positive correlation between AGEs and production of IL-2, IL-4, IL-6, and IL-1β	(307)
	MG-AGE	pro	primary human samples		increase glycolytic activity and associated with colorectal cancer progression	increase MG-AGEs levels and dicarbonyl stress, decrease GLO1 activity	(308)

Any pro-tumor effect by MG and MG-AGEs can likely be attributed to a survival mechanism; cancer adapts to withstand detrimental effects of altered metabolic flux and rather uses it to its benefit, while antitumor effects are due to overwhelming dicarbonyl stress exceeding the tumor‘s detoxification capacity (Table 1). We hypothesize this is due to the complex metabolic and physiological milieu as well as biological variation that may cause different responses to these metabolites. Therefore, the precise concentration of MG and MG-AGEs that delineates a pro- or antitumor impact is not yet clear, as there are additional biological factors to consider. Despite this, measuring blood-derived cultures of both healthy and cancer patients revealed an upregulation of MG-AGEs in those with cancer.^251^ Furthermore, the pro-cancer role of AGE/RAGE activation and its downstream signaling cascades has been well established across multiple types of cancers, showing it inhibits apoptosis^252^ and promotes autophagy,^252^ angiogenesis via VEGF,^146^ growth,^253^ inflammation,^146^ and metastasis via pathways such as AP-1, NFκB, STAT3, SMAD4, MAPK, mTOR, and PI3K.^119,254−257^

### MG and MG-AGEs in Diabetes

Given that glucose metabolism is central to MG production, MG is heavily implicated in diseases where glucose levels are elevated, particularly T1D and T2D. In addition to inducing oxidative stress and inflammation via AGE/RAGE signaling, elevated intracellular MG levels impaired cellular responses to insulin, particularly with ERK1/2 and AKT,^258^ a signaling pathway indispensable in regulating insulin sensitivity and glucose uptake.^259−261^ Thus, excess MG contributes to insulin resistance, which is characteristic of T2D.^258^ Both serum MG and MG-AGEs are significantly elevated in individuals with T1D and T2D.^208,262−264^ When measured in young patients with T1D without complications, serum MG levels were significantly higher than their control nondiabetic counterparts.^265^ These findings have been recapitulated in newly diagnosed patients with T2D, supporting a link between elevated MG levels and development of either T1D or T2D.^266^

Individuals with T1D or T2D are at a high risk of developing secondary complications. Because these complications are associated with poor glycemic control, MG and MG-AGEs are proposed to be associated with and potentially drive them through RAGE-dependent mechanisms.^267^ The association of both MG and MG-AGEs in diabetic complications such as nephropathy, cardiovascular problems, cancer, and skeletomuscular disease have been extensively covered above. In addition to these, retinopathy,^268^ neuropathy,^269^ and vascular complications^270^ are associated with MG-AGEs.^271−273^

## Measuring MG and MG-AGEs

**Figure 6:**
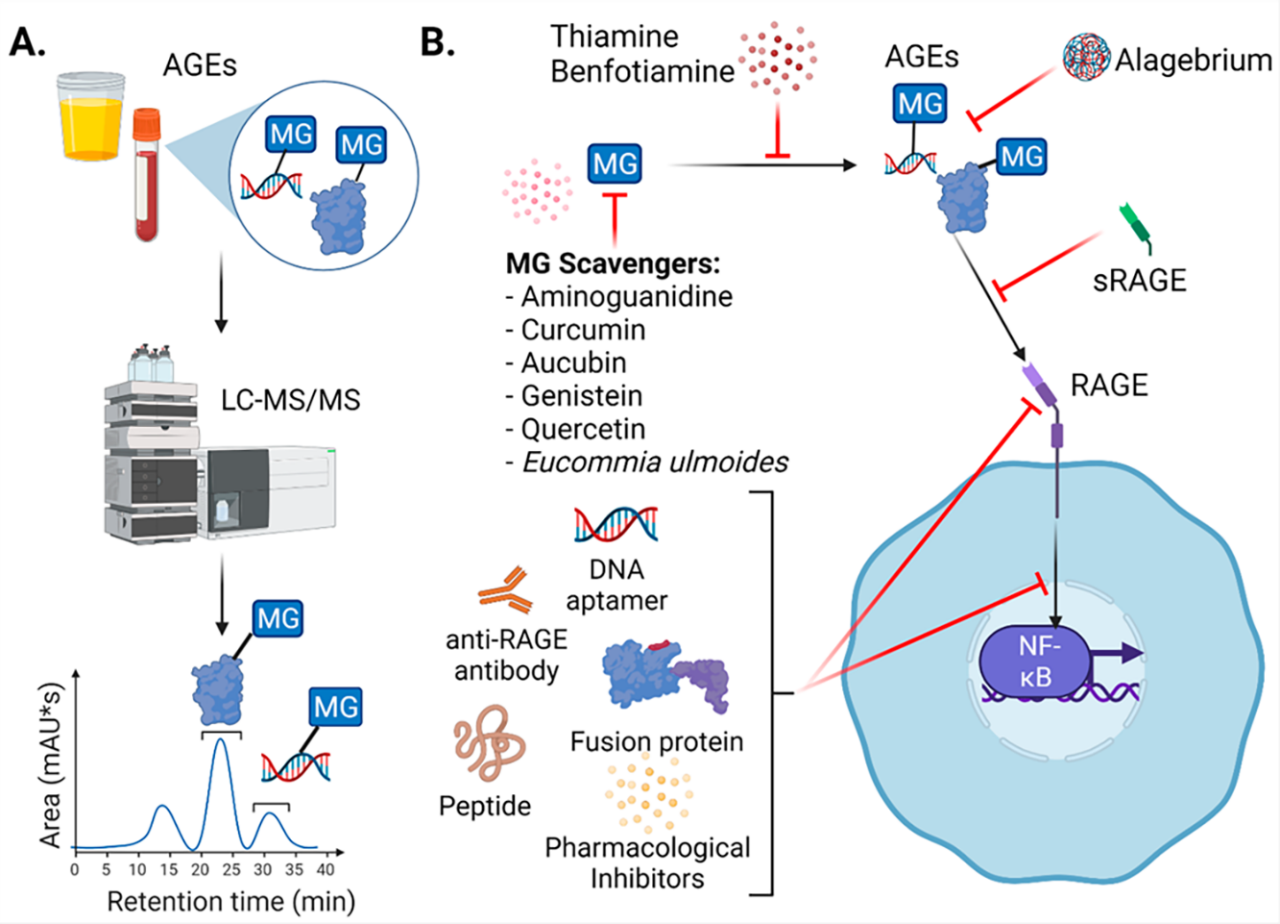
Approaches for measuring and targeting MG, MG-AGEs, and RAGE. (A) MG-AGEs can be quantified in vitro and in biological samples using LC-MS/MS. Example chromatogram is shown of the elution of MG-modified protein and DNA. (B) Molecules such as aminoguanidine and curcumin can be used to scavenge MG. MG and the formation of MG-AGEs can be targeted via scavenger compounds and using thiamine or benfoatiamine. AGEs can be targeted using sRAGE or alagebrium. RAGE activation can be inhibited using DNA aptamers, anti-RAGE antibodies, fusion proteins, peptides, or pharmacological inhibitors. Created with BioRender.com.

Direct MG quantification is difficult because of its reactivity; therefore, MG-AGEs are proposed to be more accurate indicators of MG production (Figure 6A). Rabbani and Thornalley pioneered a unique technique to detect and quantify MG using stable isotopic dilution liquid chromatography with tandem mass spectrometry (LC-MS/MS).^1^ Using this method, they achieved a limit of detection of 8 fmol MG and a limit of quantitation of 90 fmol MG.^1^ This approach has clinical applications by measuring MG in blood and tissue.^1,276^ Likewise, technologies to measure MG-AGEs have provided diagnostic and prognostic tools. Initial approaches involved the use of skin autofluorescence, a noninvasive technique that measures tissue accumulation of MG-AGEs, a technique that has proven useful in predicting development of cardiovascular disease,^277^ microvascular complications,^278^ or kidney transplant rejection.^279^ In contrast, newer methods use mass spectrometry or colorimetric and fluorometric profiling of MG-AGEs to allow for rapid measurement and analysis of clinically relevant concentrations.^278,280−283^

Quantification of MG and MG-AGEs via methods such as LC-MS/MS and ELISA support the use of MG and MG-AGEs as biomarkers for diseases such as T2D, Alzheimer’s disease, chronic kidney disease, nonalcoholic fatty liver disease, atherosclerosis, and others.^50,150,200,274,275^ MG-H1 was measured in biological samples, including aortic tissue and lens protein, with a high correlation to AGE formation.^77,284^ Taken together, this presents a novel and quantifiable class of metabolites to aid in not only predicting disease but also informing disease state and prognosis.

### Targeting MG and MG-AGEs and Preventing Their formation

The prevalence of MG and MG-AGEs during both normal and disease states makes regulating their levels an attractive target to mitigate disease severity and progression. Aside from the canonical pathways involved in MG detoxification, recent advances have generated considerable interest in approaches to selectively target MG and MG-AGEs to offset their associated damage and effects (Figure 6B, Table 2).^145^ While there are pharmacological approaches to preventing MG and MG-AGE formation and accumulation, their potential for clinical application requires additional investigation. Alternatively, nonpharmacological approaches such as diet and exercise may have utility in decreasing MG and MG-AGE formation by regulating metabolic flux.^145^

**Table 2:** Approaches to Targeting MG and/or AGEs

target	name	modality	model	effect	proposed mechanism	source
MG	aminoguanidine	scavenger	diabetic rats, human endothelial cells, rat mesangial cells	prevents AGE formation, attenuates diabetic complications in vivo	binds to carbonyl groups and converts them to nontoxic byproducts (3-amino-6-methyl-1,2,4-triazine and 3-amino-5-methyl-1,2,4-triazine)	(309−317)
MG	curcumin	scavenger	mouse blastocysts and embryonic stem cells, human mononuclear and endothelial cells, diabetic rats	decreases apoptosis and oxidative stress, mitigates MG-induced DNA damage, anti-inflammatory and antioxidant	scavenges MG by forming adducts at the 10th carbon between keto carbon groups; synergizes with aminoguanidine for increased benefit	(313,318−322)
MG	aucubin	scavenger	in vitro models and in vivo MG-injected rats	inhibits AGE formation and prevents their accumulation		(323)
MG	genistein	traps MG	in vitro	inhibits AGE formation	forms mono-MG and di-MG adducts of genistein	(324)
MG	quercetin	traps MG	in vitro	inhibits AGE formation in dose-dependent manner; traps MG and glyoxal	forms MG adducts to make mono-MGO and di-MGO adducts	(325)
MG	*Eucommia ulmoides*	promotes MG detox	in vitro and diabetic mice	inhibits AGE formation and accumulation, decreases RAGE expression, and reduces oxidative stress	upregulates Glo1 and Nrf2 pathway to increase GLO1 production and oxidative protection	(326)
AGEs	sRAGE	scavenger	human endothelial cells, diabetic and nondiabetic apoE-null mice	significant reduction of atherosclerotic lesions and inflammation, ameliorates vascular permeability		(115,161,327)
AGEs	thiamine	vitamin	human endothelial cells, bovine retinal endothelial cells	inhibits AGE formation and mitigates oxidative stress	promotes metabolism of glycolysis metabolites	(328)
AGEs	benfoatiamine	vitamin	diabetic rats, T1D and T2D patients	restores nerve conduction velocity, inhibits AGE formation, prevents diabetes-induced glycoxidation products, prevents micro- and macrovascular endothelial dysfunction, mitigates oxidative stress		(329−333)
AGEs	alagebrium	cross-link breaker	old primates and humans, diabetic rats	improves cardiac function and output, and endothelial function	removes new AGEs by separating α-dicarbonyl carbon–carbon bonds formed in cross-links	(334−338)

## Targeting RAGE

Pharmacological targeting of RAGE has been explored using antibody and small-molecule-based methods. Blocking RAGE activation by treating endothelial cells with anti-RAGE antibodies decreased the oxidative damage caused by RAGE activation, highlighting its potential as a therapeutic approach.^285^ Since then, RAGE inhibition using compounds such as FPS-ZM1 or azeliragon in a murine model of acute lung injury reduced RAGE activation and expression and decreased inflammation and damage.^286^ Similar findings have been reported in murine models of Alzheimer’s disease as well, finding that azeliragon was nontoxic in rats and helped reduced Alzheimer’s injury and progression.^287^ Additional human clinical trial evidence has supported the use of azeliragon in ameliorating the deleterious effects associated with RAGE activation, yielding promising results and demonstrating its safety profile and tolerance in humans.^288,289^ Although the precise mechanism by which azeliragon acts has not been fully elucidated, it is proposed to be a RAGE antagonist.^287^ Similar findings have been reported in studies of GM-1111, a semisynthetic glycosaminoglycan ether, which was found to inhibit interactions between RAGE and its ligands, such as S100B, HMGB-1, and CML-BSA, an AGE.^290^ In addition, other inhibitors and approaches to targeting RAGE have been developed. They are shown in Figure 6B and summarized in Table 3. However, due to RAGE’s ubiquitous nature, there is a risk of off-target toxicity and unwanted side effects that could result from indiscriminate RAGE targeting.

**Table 3:** Approaches to Targeting RAGE

type	name/nature	model	effect	source
antibody	rat antimouse monoclonal	septic mouse model	prolonged survival	(339)
	humanized monoclonal	pneumonic mouse model	prolonged survival	(340)
	rabbit polyclonal	rat liver injury model	decreased necrosis, inflammation, and fibrosis; protected from further liver injury	(341)
	goat polyclonal	Duchenne muscular dystrophy mouse model	reduced necrosis and inflammation	(342)
	rabbit polyclonal	systemic inflammation mouse model	reduced inflammation and activation of ERK1/2, p65, and IkB	(343)
	mouse monoclonal	uremic mouse model	reduces atherosclerosis	(344)
	antihuman/mouse monoclonal	neuropathic pain mouse model	attenuation of inflammation and neuropathic pain	(345)
	antimouse/rat monoclonal	lung cancer mouse model	suppressed metastasis	(346)
peptide	S100P-derived RAGE antagonist	glioma and pancreatic cancer mouse model	blocked ligands’ ability to bind and stimulate RAGE and NFkB; reduced growth and metastasis of tumors	(347)
	inhibitory peptides for RAGE signaling	SH-SY5Y and U-87MG cells	reduced neuronal cell death, inhibit invasion and migration of glioma cells	(348)
fusion protein	TAT-SAM blocks interaction between RAGE and SLP76, a critical adaptor protein for RAGE function	septic mouse model	decreases tissue damage and RAGE cytokine release and downstream signaling; prolonged survival of mice	(349)
aptamer	short DNA sequence created using SELEX	mesangial cells and diabetic nephropathy rat model	suppression of AGE-induced oxidative stress, inflammation, fibrosis, albuminuria, and podocyte damage	(350)
		HCT116 cells and colorectal cancer mouse model	suppression of RAGE/NFkB signaling, inhibition of tumor growth, decreases cell proliferation and migration	(351)
		melanoma mouse model	inhibit tumor growth, decrease AGE and RAGE expression, lowered oxidative stress and angiogenesis	(352)
pharmacological inhibitor	4,6-bis(4-chlorphenyl)pyrimidine analogue	Alzheimer’s disease mouse model	inhibition of amyloid-beta plaque accumulation, improvement of cognitive function	(353)
	pyrazole-5-carboxamide	Alzheimer’s disease mouse model	inhibition of amyloid-beta plaque accumulation	(354)
	FPS-ZM1	breast cancer mouse model	impaired tumor growth and angiogenesis, decreased inflammation, inhibition of metastasis	(355)
		Alzheimer’s disease mouse model	decreased amyloid-beta plaque accumulation in brain, suppressed inflammation, improved cognitive performance	(356)
		acute lung injury mouse model	decreased RAGE expression and inflammation, restored cell contacts and epithelium integrity	(286)
	azeliragon	acute lung injury mouse model	decreased RAGE expression and inflammation, restored cell contacts and epithelium integrity	(286)
		Alzheimer’s disease rat model	reduced AD injury, reversed neuronal damage, increased neurological function	(287)
	semisynthetic glycosaminoglycan ethers (SAGE)	in vitro models and rosacea mouse model	inhibits ligands from binding to RAGE, reduces inflammation	(290)
		secondhand smoke mouse model	decreased inflammation, RAGE signaling; increased AXL and Gas6 protein expression	(357,358)

## Conclusion

All living organisms perform metabolism, a process vital for life. Despite its necessity in sustaining life, metabolic perturbations underlie the pathology of many diseases. The mechanisms leading this are not clear, but MG, MG-AGEs, and RAGE are shown to play a critical role in many metabolism-driven diseases. Elucidation of their role in promoting disease onset and progression has been increasingly appreciated and studied as an invaluable asset to aid in our understanding of how and why certain diseases develop.

Despite many significant clinical advances in the treatment of human disease, there remains a gap in knowledge allowing us to predict and intervene to prevent or treat diseases early rather than after irreversible damage has occurred. Furthermore, there is a shortage of clinically relevant and quantifiable biomarkers of disease that would aid in the process. Current ways of predicting disease, such as the measurement of HbA1c in diabetes, only allow physicians to see a snapshot in time. On the other hand, metabolism is a process that can be continually monitored, and metabolism flux can be studied as it occurs. Therefore, we believe that the study of metabolites and their role in disease as progressors and predictors may yield a slew of novel viable biomarker candidates for clinical application. As such, further characterization of the biochemistry and interactions of MG, MG-AGEs, and RAGE is critical to study how their role in the body can be exploited for therapeutic benefit.

## Abbreviations

2-ODH: 2-oxoaldehyde dehydrogenase
AD: Alzheimer’s disease
ALDH: aldehyde dehydrogenase
AGEs: advanced glycation end products
AKT: protein kinase B
ALKBH7: Alkb homologue 7
AMOO: acetol mono-oxygenase
AR: aldose reductase
Bcl-2: B-cell lymphoma-2
BER: base excision repair
CEdG: *N*^2^-(1-carboxyethyl)-2′-deoxyguanosine
CEG: *N*^2^-(1-carboxyethyl)-guanosine
CEA: *N*^7^-carboxyethyl arginine
CEC: carboxyethyl cysteine
CEL: N^ε^-carboxyethyllysine
CKD: chronic kidney disease
cMG-dG: 1,*N*^2^-(1,2-dihydroxy-2-methyl)ethano-deoxyguanosine
dG: deoxyguanosine
DHAP: dihydroxyacetone phosphate
ERK: extracellular signal-regulated kinase
esRAGE: endogenous secretory receptor for advanced glycation end products
G3P: glyceraldehyde-3-phosphate
G3PDH: glycerol-3-phosphate dehydrogenase
G3PO: l-glycerol-3-phosphate oxidase
GK: glycerol kinase
GLO1 (human), Glo1 (murine): glyoxalase 1
GLO2: glyoxalase 2
GSH: glutathione
HMGB1: high-mobility group box-1
HPLC: high-performance liquid chromatography
HPRT: hypoxanthine phosphorylribosyltransferase
HSP: heat shock protein
JAK: Janus kinase
JNK: c-Jun N-terminal kinase
LC-MS/MS: liquid chromatography with tandem mass spectrometry
MAPK: mitogen-activated protein kinases
MEK: mitogen-activated ERK kinase
MG: methylglyoxal
MG-AGEs: methylglyoxal-derived advanced glycation end products
MG-BSA: methylglyoxal bovine serum albumin
MG-CEdG: *N*^*2*^-(1-carboxyethyl)-7-1-hydroxy-2-oxopropyl-dG
MG-H1: Nδ -(5-hydro-5-methyl-4-imidazolon-2-yl)ornithine
MG-H2: 2-amino-5-(2-amino-5-hydro-5-methyl-4-imidazolon-1-yl)pentanoic acid
MG-H3: 2-amino-5-(2-amino-4-hydro-4-methyl-5-imidazolon-1-yl)pentanoic acid
MMP: matrix metalloproteinase
MMR: mismatch repair
MODIC: lysine–arginine protein dimer induced by MG
MOLD: lysine–lysine protein dimer induced by MG
MP: myeloperoxidase
MS: methylglyoxal synthase
mTOR: mammalian target of rapamycin
NAD: nicotinamide adenine dinucleotide
NADP: nicotinamide adenine dinucleotide phosphate
NER: nucleotide excision repair
NFκB: nuclear factor kappa B
PD: Parkinson’s disease
PI3K: phosphatidylinositol 3-kinase
RAGE: receptor for advanced glycation end products
ROS: reactive oxygen species
SMAD1: SMAD family member 1
sRAGE: soluble receptor for advanced glycation end products
SSAO: semicarbazide-sensitive amine oxidase
STAT: signal transducer and activator of transcription
T1D: type 1 diabetes
T2D: type 2 diabetes
TAZ: transcriptional coactivator with PDZ-binding motif
TDH: threonine dehydrogenase
TGF-β1: transforming growth factor beta 1
TNBC: triple-negative breast cancer
TPI: triose phosphate isomerase
